# Construction and sustained release of konjac glucomannan/naringin composite gel spheres

**DOI:** 10.3389/fnut.2022.1123494

**Published:** 2023-01-19

**Authors:** Liping Dao, Siyang Chen, Xiangyun Sun, Wenyuan Pang, Hengzhe Zhang, Jun Liao, Jiqiang Yan, Jie Pang

**Affiliations:** ^1^College of Food Science, Fujian Agriculture and Forestry University, Fuzhou, China; ^2^College of Computer and Information, Fujian Agriculture and Forestry University, Fuzhou, China

**Keywords:** konjac glucomannan, naringin, sodium alginate, mesoporous silicon dioxide, sustained release system

## Abstract

**Objective:**

To improve the bioavailability of active substances and reduce the toxic and side effects on the human body, natural biological macromolecules are used to load active substances and control their release speed in different environments of the human body. In this study, mesoporous silica (MSN) was combined with konjac glucomannan (KGM) and sodium alginate (AC) to prepare pH-sensitive konjac glucomannan/sodium alginate–mesoporous silica loaded naringin gel spheres (KS/MSN). On this basis, the structure, morphology, and release properties of the composite gel spheres were characterized. The results showed that the cumulative release rates of both simulated gastric fluid (SGF) and Simulated colonic fluid (SCF) were lower than that of simulated small intestinal fluid (SIF), which indicated that the prepared composite gel spheres were pH-sensitive to SIF and obtained the best release rate of about 70% under SIF environment.

**Methods:**

The pH-sensitive konjac glucomannan/sodium alginate composite gel spheres (KGM/SA) were prepared by combining inorganic nano-materials mesoporous silica (MSN) with natural macromolecular polysaccharides konjac glucomannan (KGM) and sodium alginate (SA) and characterized.

**Results:**

The results showed that there was a process of ionic crosslinking and entanglement between konjac glucomannan (KGM) and sodium alginate (SA). Naringin (NG) and mesoporous silica (MSN) were successfully compounded and had good compatibility. The gel microstructure diagram showed that the addition of MSN improved the gel properties of KGM, and KGM and SA gel spheres (KGM/SA) had good compatibility with mesoporous silica/naringenin nanoparticles (NG/MSN). The study of the simulated digestive environment of the gastrointestinal release medium showed that Konjac glucomannan/sodium alginate-mesoporous silica loaded naringin gel spheres (KS/NM) composite gel spheres had the best slow-release effect and the highest final-release completion degree in SIF. The release of NG from KS/NM composite gel spheres showed a slow upward trend. The results showed that KS/NM composite gel spheres were pH-sensitive.

**Conclusion:**

The KS/NM composite gel spheres showed obvious pH sensitivity to the release of NG, and the gel spheres had a good sustained release effect on NG.

## 1. Introduction

**FIGURE 1:**
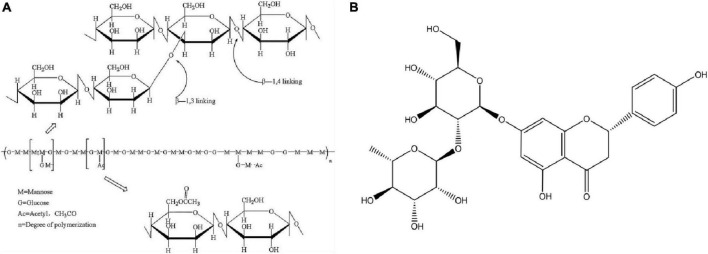
The chemical structure of KGM **(A)** and NG **(B)**.

In recent years, natural polymer sustained-release carriers have become the focus of attention because of their excellent biocompatibility, degradability, and abundant sources (1). Konjac glucomannan (KGM) is a functional polysaccharide whose chemical structure is shown in Figure 1A (2). KGM is a good gel and thickener in food because of its edible and stable physicochemical properties and has been used to control body weight and intestinal health (3). In addition, KGM is also widely used in packaging and biomedical fields, and has been used to prepare sustained-release carrier systems. KGM-based sustained-release materials, such as hydrogel film microspheres, have been widely studied (4–6). Based on the excellent physical and chemical properties of KGM, the gel based on KGM can drive the release of active substances, and its good swelling performance can obviously improve the release of functional compounds. But the pure KGM gel has the problem of strong hydrophilicity and poor mechanical properties, which cannot protect the active substances and slow-release effect (7, 8). Sodium alginate (SA) and its aqueous solution generally have high viscosity and can be used in food industry to promote food quality technology, such as thickeners, stabilizers, emulsifiers, and so on. With the development of sustained-release systems and biomedical materials. SA can be used as a biomagnetic, conductive and pH-sensitive gel carrier material with different functions, and is widely used to immobilize or embed active small molecules, proteins and drugs in the form of hydrogels, aerogels, and microcapsules (9, 10). Because of these excellent properties, SA has been widely used in the food industry, tissue engineering, biomedicine, and other fields.

Naringin (NG) is a kind of flavonoid extracted from citrus fruits. Its chemical structure is shown in Figure 1B. It is also an active ingredient in Chinese herbal medicine. It has many biological activities, such as broad-spectrum antibacterial, anticancer, hypolipidemic, and hypoglycemic (11–13), and it can be used as a potential drug for regulating intestinal flora. However, NG is insoluble in water and has poor fat solubility. If the dosage is too high or too low, its maximum effect cannot be exerted. Therefore, the water solubility of NG is enhanced to ensure its bactericidal activity at the optimum concentration, thereby improving its therapeutic effect. It is of great significance to maximize the utilization of NG (14). In this regard, we used mesoporous silica (MSN) as its carrier. As a nano-inorganic material, MSN has the advantages of a large specific surface area, good biocompatibility, high chemical stability, and low toxicity. It has been extensively researched and applied (15–17), and through experimental design, the active substance delivery process can be realized, or there will be no reaction in the normal part, and the induced release can only be achieved in a certain part.

In this study, gel spheres with encapsulation effect were prepared using KGM and SA as raw materials, and used to encapsulate NG/MSN, and finally obtained KS/NM. Furthermore, the protective and release effects of KGM/SA as gel carriers were studied by using NG as a biologically active material model, and the release behavior and release mechanism of NG in different simulated media were discussed. It lays the foundation for the slow release of active substances and has great significance for enhancing the release ability and improving the curative effect of active substances.

## 2. Materials and methods

### 2.1. Materials and instruments

Konjac glucomannan (purity ≥ 90%) was used without any further purification, purchased from Sichuan Ping Shan Fish Spring White Magic Potato Co. (Pingshan, China). Sodium Alginate was purchased from Yuanye Biotechnology Ltd. (Shanghai, China). Mesoporous silica and Naringin were purchased from Sigma-Aldrich Reagent Co. Ltd. (Shanghai, China).

Magnetic agitator (SHJ-6AB), electronic balance (ME204TE/O2), freeze dryer (SCIENTZ-10N), shaking table (XY-ZDS-C), high-performance liquid phase (S22H), ultrasonic cleaning machine (DHG-9030), oven (PHS-3E), and pH meter (SHJ-6AB).

### 2.2. Preparation of MSN/NG nanoparticles

Prepared with reference to the methods of Del et al. (18) and Yuan et al. (1).

2 g of MSN powder was weighed and immersed in NG/methanol solution, and the mixture was placed in the beaker. The mixture was ultrasonicated for 30 min under negative pressure and stirred magnetically for 12 h at room temperature to reach adsorption equilibrium. In order to reach the maximum loading in the MSN channel. Finally, the solvent was removed by evaporation and the powder was dried at 90°C. The MSN loaded with NG were obtained by evaporation to remove the solvent and drying at 90°C.

### 2.3. Preparation of NG gel spheres supported by KS/MSN

**FIGURE 2:**
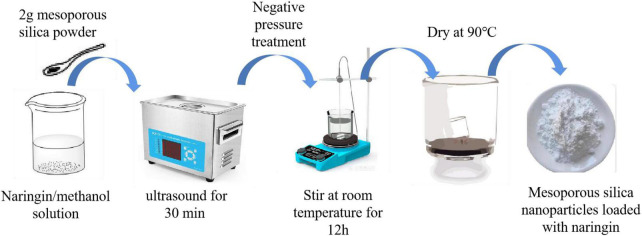
Preparation of MSN/NG nanoparticles.

The preparation method was slightly modified according to Hezma et al. (19). As shown in Figure 2, the volume fraction of 1% NG/MSN was ultrasonically dispersed in an aqueous solution to obtain a 1% volume fraction of NG/MSN suspension. Add a certain proportion and a certain amount of KGM/SA polysaccharide mixed with the powder with a total concentration of 1.5% to the suspension, stir at 60°C for 4 h and place it at room temperature until there are no bubbles. At a constant pump speed, the polysaccharide-mixed solution was added dropwise to the CaCl_2_ solution with a mass fraction of 3%. After the cross-linking was uniformly solidified for 30 min, it was rinsed with deionized water several times, and the excess water on the surface of the gel spheres was blotted and freeze-dried.

### 2.4. Structural characterization

#### 2.4.1. X-ray diffraction analysis (XRD)

The symmetry and order of the pore structure of the sample were analyzed. The XRD spectra of the sample were scanned in the range of small angle 2θ = 0.5–5° and wide angle 2θ = 2–150°.

#### 2.4.2. Porous physical adsorption analysis (BET)

Adsorption-desorption isotherms of nitrogen were measured by Konta EVO porous physical adsorption instrument at liquid nitrogen temperature (−196°C); The specific surface area is calculated according to the BET formula at the relative pressure P/P_0_ = 0.05–0.35; According to BJH (Barrett-Joyner-Halenda) equivalent cylindrical model, the pore size distribution is calculated from the adsorption and desorption branches of the adsorption isotherm; The pore volume is determined by the amount of N_2_ adsorbed at or near the relative pressure *P*/*P*_0_ = 0.95.

#### 2.4.3. Field emission scanning electron microscope (SEM)

The microstructure of the samples was characterized by SEM. After drying the sample, stick the sample on the sample table with conductive adhesive, put it in an instrument, coat the sample under a vacuum, put the gold plating layer under a microscope, and scan it under 20 kV accelerated voltage.

#### 2.4.4. High-resolution transmission electron microscope (HRTEM)

The sample of a certain mass was dispersed in absolute ethanol by ultrasonic wave, and the sample of uniform dispersion was dripped on the copper mesh coated with carbon film and dried. Then the copper mesh with the sample was observed under a transmission electron microscope, and the acceleration voltage was 80 kV at room temperature.

#### 2.4.5. Fourier transform infrared spectroscopy (FT-IR)

The infrared spectrum is measured by a vertex 70 Fourier infrared spectrometer produced by Bruker Company, Germany. After the dry sample powder is fully ground in the agate mortar, the proper amount of dry KBr powder is added, ground evenly, and pressed into the infrared spectrometer for scanning. The scanning range is 400–4000 cm^–1^ with a resolution of 4 cm^–1^ and 32 scans.

#### 2.4.6. Simultaneous thermal analysis (TG-DTA)

TG-DTA uses STA-8000 TG-DSC synchronous thermal analyzer made by PE Company of USA. The thermal behavior of the sample was obtained by increasing the temperature of the sample from 25 to 800°C at a heating rate of 10°C/min under nitrogen gas flow.

### 2.5. Sustained release experiment

#### 2.5.1. Preparation of release medium solution

Simulated gastric fluid (SGF, pH 1.2 hydrochloric acid solution): 8.33 mL of 0.1 mol/L hydrochloric acids, add deionized water to the mark, store at room temperature, and set aside.

Simulated small intestinal fluid (SIF, pH 6.8 phosphate buffer): dissolve 2.72 g, KH_2_PO_4_, and 0.32 g potassium hydroxide in a small amount of deionized water, transfer to a 1000 mL volumetric flask, add deionized water to the volume, and store at room temperature, set aside.

Simulated colonic fluid (SCF, pH 7.4, phosphate buffer): Dissolve 8.00 g NaCl, 0.20 g KCl, 1.44 g Na_2_HPO_4_, and 0.24 g KH_2_PO_4_ in 800 mL deionized water; adjust the pH to 7.4 with 1 mol/L HCl; transfer into a 1000 mL volumetric flask; add deionized water to the mark to achieve a constant volume; store at room temperature; and set aside. The above-mentioned medium solution was prepared (20).

#### 2.5.2. Liquid chromatographic conditions

Detection by chromatographic column Sun fire C18 (4. 6 × 250 mm, 5 μm). The mobile phase of chromatography was methanol (∼0.1%) and phosphate buffer solution (40:60), the column temperature was 30°C, the flow rate was 1.2 mL/min, the injection volume was 10 μL, and the detection wavelength was 282 nm.

#### 2.5.3. Determination of NG standard curve

The amount of 1 mg of NG was weighed and dissolved in 100 mL methanol as the control sample solution. Dilute 0.2, 0.4, 0.6, 0.8, 1.0, 1.2 ml of the sample solution in 10 ml volumetric flasks with ethanol until the scale is shaken well. The concentration of NG was 0.2, 0.4, 0.6, 0.8, 1.0, 1.2 μg/ml and the peak area at 282 nm was measured to establish the standard curve regression equation.

Determination of NG Standard Curve in SCF (pH 7.4): Precise Weighing 1.00 mg NG Dissolved in SCF and Diluted in 10 mL. Volumetric Flask to Scale as Mother Liquor Transfer the mother liquor from 0.2, 0.4, 0.6, 0.8, 1.0, 1.2 to 10 ml volumetric flasks, and then dilute with SCF to scale to prepare NG solution with a concentration of 0.2, 0.4, 0.6, 0.8, 1.0, 1.2 μg/ml as standard solution. Using SCF as a blank solution, the peak area of standard solution at 282 nm was measured by high-performance liquid chromatography, and the standard curve between peak area and NG concentration was obtained.

The standard curve method for the determination of NG in SGF (pH 1.2) and SIF (pH 6.8) is the same as that in SCF.

Each group is parallel three times. According to the measured absorbance, the standard curve for methanol solution is y = 21110x − 355.62 with a correlation coefficient *R*^2^ of 0.9998, the standard curve for SGF is y = 14198x + 216.36 (*R*^2^ = 0.9995), the standard curve for SIF is y = 11016x + 236.89 (*R*^2^ = 0.9986), and the standard curve for SCF is y = 14785x − 170.11 (*R*^2^ = 0.9995).

#### 2.5.4. Determination of load

The content of NG in MSN was determined by high-performance liquid chromatography (HPLC). 1.00 mg sample was dissolved in 10 mL methanol and shaken for 24 h at room temperature. The dissolved amount of active substance was determined by high-performance liquid chromatography. The NG loading in MSN was determined by calculating the active substance content (DC) and encapsulation efficiency (EE) (21).


(1) $$DC\%=\frac{{\text{m}}_{1}}{{\text{m}}_{2}}$$

Where *m*_1_ and *m*_2_ represent the measured NG content and the active carrier mesoporous silica content, respectively.


(2) $$EE\%=\frac{m_{3}}{m_{4}}$$

Where *m*_3_ and *m*_4_ represent the content of NG and the total amount of NG added to the active material mesoporous silica, respectively.

#### 2.5.5. Release process

The release behavior of composite gel spheres was investigated by dialysis detection. A certain amount (∼40 mg) of composite gel spheres were weighed accurately and then dispersed in a dialysis bag (molecular weight cut-off 8000–14000 Da) with 5 mL SCF release medium and sealed. The dialysis bag was placed in 95 mL corresponding release medium at 200 r/min, and the sustained release experiment of active substances was carried out in a constant temperature water bath at 37°C. 1 mL corresponding release medium was accurately removed at 1.0, 2.0, 3.0, 4.0, 5.0, 6.0, 7.0, 8.0, 9.0, 10.0, 11.0, 12.0, and 24.0 h after the start of the experiment, and 1 mL fresh corresponding release medium at 37°C was immediately added to the experimental solution. The sample was filtered by 0.22 μm microporous membrane, and its peak area was measured by high-performance liquid chromatography at 282 nm wavelength. The concentration and cumulative release rate were calculated according to the standard curve, and the cumulative release rate-time curve was drawn. The sustained release experiment of active substances was repeated three times.

The method for evaluating the SGF (pH 1.2) and SIF (pH 6.8) active substance release are the same as the above.

#### 2.5.6. Determination of cumulative release

The peak area of samples taken at each time point in the *in vitro* release experiment was measured at λ = 282 nm. The content of active substances in the release medium was calculated by standard curve, and the cumulative release rate of active substances was calculated according to formula (3).

Drawing the relationship curve between cumulative release rate and time.


(3) $$Q=\frac{C_{n}\times V_{0}+\left(C_{1}+C_{2}+C_{3}\cdots +C_{n-1}\right)\times V}{W}$$

Where *Q* is the cumulative release rate (%) of the active substance at the nth sampling point, *C*_*n*_ is the concentration of an active substance at the nth sampling point, *V*_0_ is the volume of release medium, i.e., 100 mL, *V* is the sampling amount, i.e., 1 mL.

#### 2.5.7. *In vitro* release kinetics analysis

In recent years, with the in-depth study of the release systems, great progress has been made in analyzing release data by using *in vitro* release curve fitting and common release kinetic models. At present, there are more than 10 mathematical models for curve fitting. In this experiment, three models commonly used to fit curves at present are mainly selected (22–24):

(1) The zero-order release kinetics model is expressed as follows:


(4) $$Q_{\text{t}}=\frac{M_{t}}{M_{\infty }}=kt$$

Where *Q*_*t*_ represents the cumulative drug release rate at time t, *M*_*t*_ and *M*_∞_ represent the cumulative drug release amount *k* at time t and time ∞, respectively, which is the zero-order release coefficient;

(2) The expression of the first-order release kinetic model is:


(5) $$Q_{t}=\frac{M_{t}}{M_{\infty }}=\left(1-e^{-kt}\right)$$

Where *Q*_*t*_ represents the cumulative drug release rate at time t, *M*_*t*_ and *M*_∞_ represent the cumulative drug release amount *k* at time t and time ∞, respectively, which is the zero-order release coefficient;

(3) The expression of the Higuchi model is:


(6) $$Q_{\text{t}}=\frac{M_{t}}{M_{\infty }}=kt^{1/2}$$

Where *Q*_*t*_ represents the cumulative drug release rate at time t, *M*_*t*_ and *M*_∞_ represent the cumulative drug release at time t and time ∞, respectively, and *k* is the Higuchi diffusion coefficient Higuchi model is mainly used to fit the drug release process based on the Fick diffusion theorem. It is also one of the most commonly used drug release models at present.

In this experiment, the release rule of NG in KS composite gel spheres was investigated by using Origin software. The release data were fitted with the above three different release kinetic models, and the release characteristics of composite gel spheres *in vitro* were analyzed according to the fitting results.

## 3. Results and discussion

### 3.1. X-ray diffraction analysis (XRD)

**FIGURE 3:**
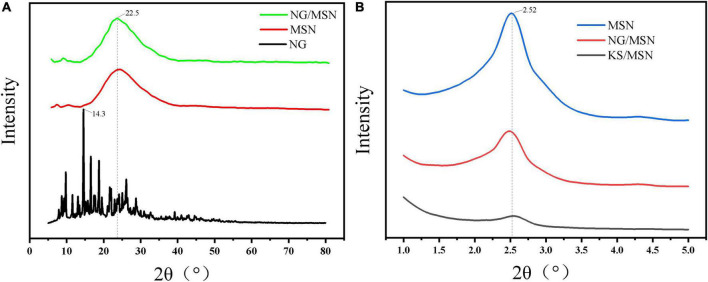
X-ray diffraction analysis (XRD) images of NG, MSN, and NG/MSN in wide-angle mode **(A)** and MSN, NG/MSN, and KS/MSN in small-angle mode **(B)**.

Figure 3 is the XRD diffraction pattern of NG nanoparticle gel spheres used to explore the internal crystal structure of KS gel. Figure 3A shows the wide-angle XRD diffraction peaks of MSN, NG, and NG/MSN. NG has an obvious sharp peak at about 14.3°. The diffraction peak trends of NG/MSN and KS/MSN are similar (25). The spectra show a wide peak peculiar to amorphous samples, but no NG diffraction peak peculiar to the crystalline phase. The absence of the drug crystalline phase indicates that NG is adsorbed in an amorphous state in the mesopore of the carrier (26), which indicates that there is a good physical interaction between them. NG may be adsorbed in the pores of MSN or polysaccharide coated on the surface of MSN The main problem of drug solubility is that the crystalline state has higher lattice energy. The thermodynamic properties (enthalpy, entropy, and free energy) of the amorphous state are higher than those of the corresponding crystalline phase, which makes amorphous solids easier to crystallize (27). The migration of nano-restricted molecules in the pores of monodisperse silica makes the amorphous phase of drugs remain stable after adsorption in the carrier. Wide-angle XRD was used to characterize the presence of NG in the MSN carrier. Low-angle XRD spectra are sensitive to pore filling, and the intensity of characteristic peaks of loaded materials is lower than that of pure materials. The main characteristic peaks in the range of 2–2.8° appeared in the XRD spectra of all materials, indicating that the MSN structure is intact (28).

### 3.2. Porous physical adsorption analysis (BET)

The structural characteristic data of samples obtained by the BET analyzer are shown in Table 1. Compared with pure MSN, the sharp decrease in specific surface area and pore volume of the other two kinds of MSN indicates that NG fills the mesopores of MSN. The specific surface area and pore volume of MSN decreased from 1637.159 m^2^/g, 1.047 cm^3^/g to 705.38 m^2^/g, 0.481 cm^3^/g, respectively, indicating that NG might be adsorbed inside and on the surface of MSN.

**TABLE 1:** Nitrogen stripping absorption results parameters.

Sample	Surface area (m^2^/g)	Pore volume (cm^3^/g)	Pore diameter (nm)
MSN	1637.159	1.047	2.385
NG/MSN	705.38	0.481	2.385
KS-NM	25.766	0.033	1.624

### 3.3. Field emission scanning electron microscopy (SEM)

**FIGURE 4:**
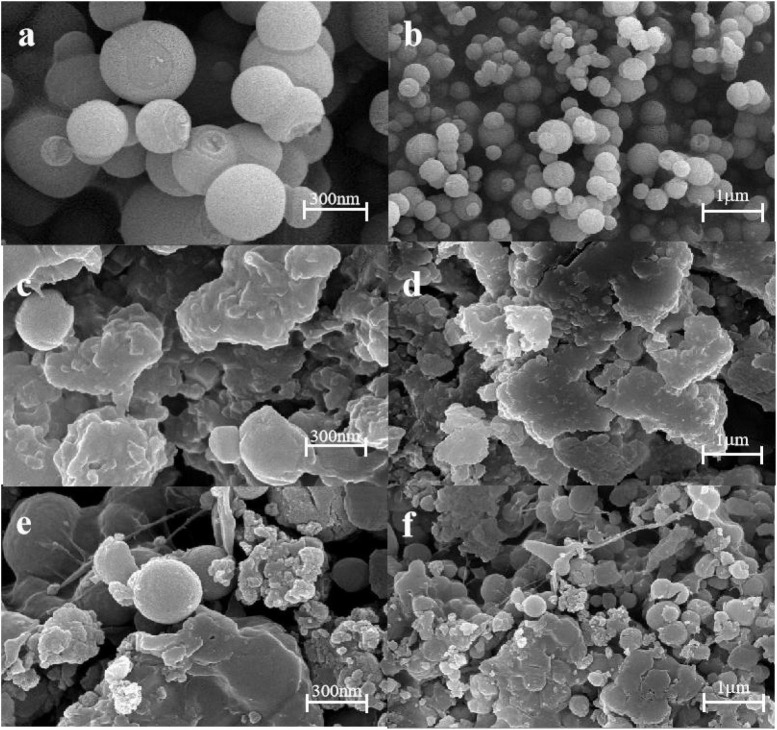
Scanning electron microscope (SEM) images of MSN **(a,b)**, KS **(c,d)**, and KS/MSN **(e,f)**.

Scanning electron microscopy was used to investigate the microscopic morphology of KGM/SA gel. The surface morphology of MSN granular gel spheres was observed by scanning electron microscope to study its microstructure. It can be observed from Figures 4a, b that the silica nanoparticles have a spherical structure. Figures 4c, d shows that the surface of KS composite gel is uniform and smooth without cracks or pores. It can be seen from Figures 4e, f that the surface of KS/MSN gel spheres became rough, and the particles were uniformly distributed after the addition of MSN. This phenomenon is attributed to the existence of MSN nanoparticles in the gel matrix. The results show that KS has good compatibility with MSN (29).

### 3.4. High-resolution transmission electron microscope (HRTEM)

**FIGURE 5:**
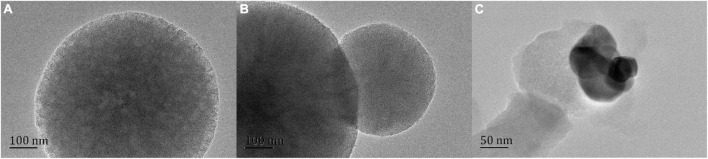
Scanning electron microscope (SEM) images of MSN **(A)** and NG/MSN **(B)**, and KS-NM **(C)**.

Further analysis of mesoporous silica structure by transmission electron microscope. As shown in Figure 5, HRTEM images clearly show a highly ordered mesoporous network, which can indicate that NG adsorption will not destroy the mesoporous structure of MSN (30). In addition, there is no agglomeration phenomenon, which indicates that NG is orderly and uniformly dispersed in MSN pores.

### 3.5. Fourier transform infrared spectroscopy (FT-IR)

**FIGURE 6:**
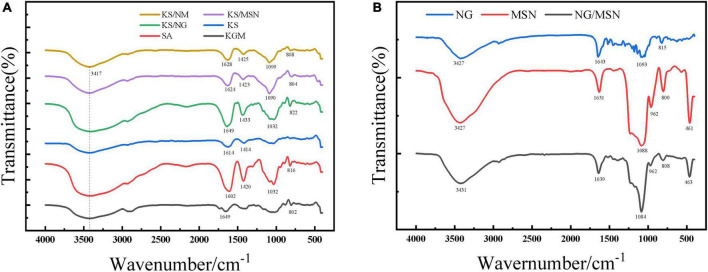
Infrared spectra of KGM, SA, KGM/SA, KS/NG, KS/MSN, KS/NM **(A)** and NG, MSN, NG/MSN **(B)**.

Fourier transform infrared spectroscopy spectra are used to detect the functional groups in the reaction mixture. To study the molecular interaction among composite gel, MSN, and NG, FT-IR spectra of samples are recorded, which are usually used to identify the functional groups in the structure of chemical substances. FT-IR images of KGM, SA, KGM/SA, KS/NG, KS/MSN, KS/NM NG, MSN, and NG/MSN are shown in Figure 6. The typical spectra of MSN are as follows: the strong peaks in the 455–470, 750–860, and 1,000–1,100 cm^–1^ regions are attributed to the vibrating, stretching, and bending forms of Si-O-Si bonds. The broad peak at 950–970 cm^–1^ is the Si-OH group of silanol (31, 32). The absorption peak at 3417 cm^–1^ is derived from the -OH stretching vibration absorption peak of KGM. Compared with MSN, the signal of NG/MSN at 1,084 cm^–1^ is weaker, which indicates that hydrogen bonding interaction is formed between hydroxyl groups in NG and silanol hydroxyl groups in MSN, which leads to the change of intensity signal of the characteristic peak. Flavonoids have polyhydroxy groups, so signal peaks appear at 3,000–3,500 cm^–1^ in FTIR spectra (33). The FT-IR spectra of NG showed that the characteristic peak of the OH group was at 3,427 cm^–1^, and the FTIR spectra of the NG/MSN mixture showed peaks at 3,431, 1,639, and 1,084 cm^–1^, while the characteristic peak of NG at 2,700 cm^–1^ remained, which indicated that NG and MSN were successfully combined. In addition, the drug peaks in the two spectra have little change, which shows that NG and MSN have good compatibility (34), and similar results have been reported (35).

In the FT-IR spectra of SA, the peaks at 1,602 and 1,420 cm^–1^ are due to the asymmetric and symmetric stretching vibrations of carboxyl groups, respectively (36). When KGM is added, the peak shift from 1,602 to 1,614 cm^–1^. These two characteristic peaks are of great significance for the study of the ionic crosslinking process. The research shows that KGM deacetylation strengthens the hydrogen bond interaction, which can promote the strength and cohesion of microspheres (37). Compared with KS, KS/MSN, and KS/NG microspheres have asymmetric-COO oscillation peaks shifting to higher wavenumber, 1,614–1,649 cm^–1^ and 1,614–1,624 cm^–1^, respectively, and the symmetric-COO oscillation peaks shifting from 1,414 to 1,423 cm^–1^ (38). These changes are attributed to the hydrogen bonding between the OH of KGM and MSN and the C = O of SA. It can be speculated that KS blends promote intermolecular interaction through hydrogen bonding (39). It is worth noting that no additional characteristic peaks were observed in the composite gel sphere KS/NM, indicating that no covalent bonds were detected between NG/MSN and KS. In addition, several characteristic peaks shifted slightly, so the interaction between NG/MSN and KS may be a physical reaction of hydrogen bonding (40), which indicates that KS and NG/MSN are well recombined.

### 3.6. Synchronous thermal analysis (TG-DTA)

**FIGURE 7:**
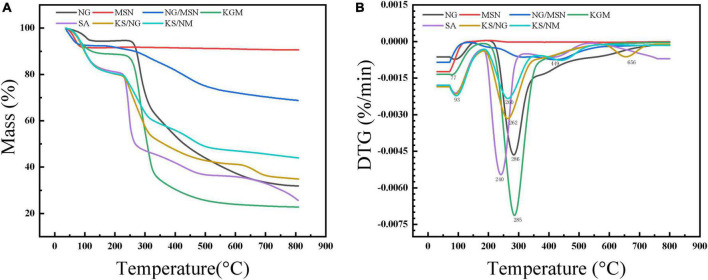
TGA curves **(A)** and DTG curves **(B)** of NG, MSN, NG/MSN, KGM, SA, KS/NG, and KS/NM.

The thermogravimetric analysis investigated the thermal stability of NG, nanoparticles, and gel spheres. It can be seen from Figure 7, the thermogravimetric analysis curves of MSN indicated that a weight loss of about 10% of the sample could be observed in the temperature range from the onset temperature to about 100°C. This is mainly related to the release of physisorbed water molecules. At 150°C, the weight of NG starts to decrease significantly due to the decomposition of NG, and the temperature from 100 to 800°C has no significant effect on the weight of MSN, reflecting the remarkable thermal stability (21, 30).

The thermogravimetric analysis curve of MSN showed that a weight loss of about 10% of the sample was observed in the temperature range from the starting temperature to about 100°C. This was mainly related to the release of physically adsorbed water molecules. At 150°C, the weight of NG started to decrease significantly due to the decomposition of NG. After MSN was mixed into the composite gel spheres, the degradation mode changed significantly. In the second stage, the degradation degree was significantly delayed due to the low bound water content and the MSN interaction of the gel spheres. Therefore, mesoporous silicon slightly improves the thermal stability of composite gel spheres in the degradation stage (21).

### 3.7. Loading rate analysis of NG nanoparticles

The actual concentration of NG was determined by high-performance liquid chromatography. We obtained the content of each substance, as shown in Table 2. According to formula (1), the loading rate of NG on mesoporous silica was 25.8%. HPLC determines the peak area at 282 nm of the NG/MSN shaker. The concentration of NG in the supernatant can be calculated according to the standard curve of NG. According to formula (2), the entrapment efficiency of MSN for NG was 68.9%.

**TABLE 2:** The NG content (m_1_, m_3_), the active carrier mesoporous silica content (m_2_), and the total amount of NG added to the active material mesoporous silica (m_4_).

	m_1_	m_2_	m_3_	m_4_
Content (μg/ml)	0.4134 ± 0.04	1.6023 ± 0.18	0.4134 ± 0.07	0.6003 ± 0.11
Loading rate (%)	25.8 ± 0.03		/58.28	
Entrapment efficiency (%)	/		68.9 ± 0.06	

### 3.8. Analysis of release law of NG-loaded composite gel spheres *in vitro*

**FIGURE 8:**
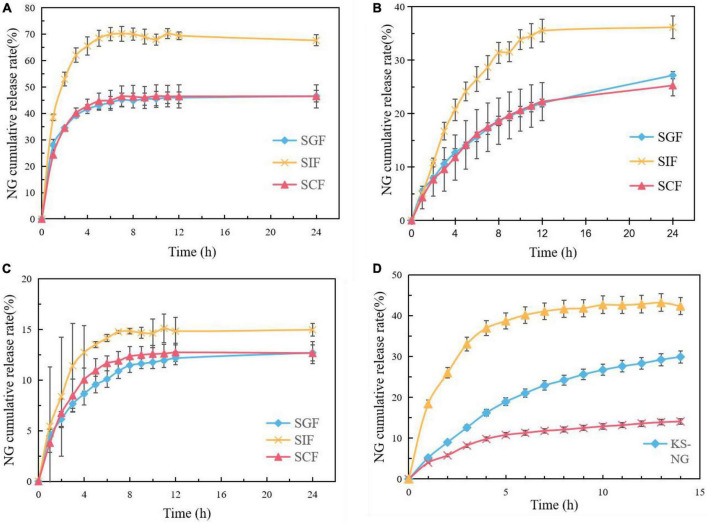
Release profiles of NG from NG/MSN at SGF, SIF, and SCF **(A)**, release profiles of NG from KS/NG at SGF, SIF, and SCF **(B)**, release profiles of NG from KS/NM at SGF, SIF, and SCF **(C)**, release profiles of NG from NM, KS/NG, and KS/NM in SGF for 2 h and subsequently in SIF **(D)**.

The release behavior of NG in SGF, SIF, and SCF was studied by *in vitro* release test, and the results are shown in Figure 8. It can be seen that the cumulative release rates of SGF and SC are lower than those of SIF, which indicates that the prepared composite gel spheres are pH-sensitive to SIF and obtain the best release rate in the SIF environment.

The cumulative release rate of NG from NG/MSN nanoparticles in SIF was about 70%. NG/MSN nanoparticles released 40% in the first H, which was much faster than that from KS gel spheres. About 35% of NG were released from KS gel spheres due to the lack of protection between NG and MSN. NG was only physically adsorbed inside MSN and the release rate was faster on the surface of MSN. The cumulative release rate of NG from KS/MSN gels in SIF was only 11% in the first 4 h and only 15% in the second 12 h. This is due to the interaction between KS and NG/MSN, which slows down the release rate of NG. It can be seen from Figure 8 that the release of NG is incomplete and the overall release rate is low. The possible reasons are as follows: the amount of NG added in the early stage is small and the release amount is not obvious; A large number of hydrogen bonding interactions between the silanol groups in the pores of mesoporous silica and NG make NG stably loaded in mesoporous silica, and because it is not a true digestive tract, it leads to the incomplete release of NG.

By comparing the smaller pH difference between SGF and SIF, the optimum pH conditions for NG release from KGM composite gel microspheres can be determined more accurately. It can be seen from Figure 8D that the slope of the cumulative release curve of KS/MSN gel spheres is lower than that of KS gel spheres and NG/MSN nanoparticles, which shows that KS/MSN gel spheres have an obvious slow release effect on NG.

### 3.9. Release kinetics analysis of composite gel spheres

Using Origin software, the experimental data of three groups of gel spheres were analyzed and fitted according to the zero-order kinetic function equation, the first-order kinetic function equation, and the Higuchi equation, respectively. The experimental results are shown in the Table 3.

**TABLE 3:** Fitting results of KS/NM gel spheres for different models release behavior at SGF, SIF, and SCF.

The kind of release medium solution	Fitting model	Fitting equation	*R* ^2^
SGF	Zero-order	*Q*_*t*_ = 0.44t + 5.99	0.5265
	First grade	*Q*_*t*_ = 12.29 (1 − e^–0.32t^)	0.9883
	Higuchi equation	*Q*_*t*_ = 2.78t^0.5^+ 2.42	0.8488
SIF	Zero-order	*Q*_*t*_ = 8.64t + 0.47	0.3635
	First grade	*Q*_*t*_ = 15.09 (1 − e^–0.45t^)	0.9971
	Higuchi equation	*Q*_*t*_ = 3.22t^0.5^+ 4.24	0.7130
SCF	Zero-order	*Q*_*t*_ = 6.74t + 0.44	0.4190
	First grade	*Q*_*t*_ = 12.89 (1 − e^–0.37t^)	0.9990
	Higuchi equation	*Q*_*t*_ = 2.87t^0.5^+ 2.91	0.7604

From the fitting results, it can be found that the goodness of fit *R*^2^ of the experimental data of gel spheres release in different release media is the largest when the first-order kinetic equation is used to fit the experimental data of gel spheres release in different release media. Therefore, the goodness-of-fit of the first-order kinetic equation is better than that of other equations when fitting the release data of three kinds of gel spheres in different release media.

## 4. Conclusion

In this study, the KS/NM composite gel spheres with pH sensitivity were prepared by combining MSN with natural polymer polysaccharide KGM and SA. The protective and release effects of KGM/SA as gel carriers were studied by using NG as a biologically active substance model, and the release behavior and release mechanism of NG in different simulated media were discussed.

Through structural characterization, it was found that NG and MSN were successfully compounded and interacted well. There was an ionic cross-linking and entanglement process between KGM and SA molecules. The microstructure of the gels showed that the addition of MSN improved the gel properties of KGM, and KS had good compatibility with NG/MSN. There were hydroxyl and acetyl groups in the gel network of KS, and MSN showed porous structure. KS composite gel spheres can be loaded with active ingredients and used in sustained release systems. At the same time, MSN has good absorption ability for active substances. KS/MSN composite gel spheres could be used as a candidate material for active substance delivery. Under simulated normal body temperature conditions (37°C), the release amount and release rate of composite gel spheres in pH 6.8 simulated media solution were significantly higher than those of other pH simulated media solutions. The KS-NM composite gel spheres showed the best slow release effect in SIF environment, and the overall released amount of NG showed a slow increasing trend with the highest final release amount. The results of the release experiments at different pH values showed that the composite gel spheres were pH sensitive. This study provided technical support for the development of KGM and the application of KGM-based porous gels in sustained release systems.

## Data availability statement

The original contributions presented in this study are included in the article/supplementary material, further inquiries can be directed to the corresponding authors.

## Author contributions

LD, SC, and XS: conceptualization, data curation, formal analysis, methodology, validation, visualization, writing—original draft and review and editing. WP, HZ, and JL: conceptualization, formal analysis, methodology, visualization, and writing—review and editing. JY: conceptualization, formal analysis, project administration, supervision, and writing—review and editing. JP: conceptualization, funding acquisition, project administration, supervision, and writing—review and editing. All authors contributed to the article and approved the submitted version.
